# Job-Deadline-Guarantee-Based Joint Flow Scheduling and Routing Scheme in Data Center Networks

**DOI:** 10.3390/s24010216

**Published:** 2023-12-30

**Authors:** Long Suo, Han Ma, Wanguo Jiao, Xiaoming Liu

**Affiliations:** 1 College of Information Science and Technology, Nanjing Forestry University, Nanjing 210037, China; wgjiao@njfu.edu.cn (W.J.); lxm@njfu.edu.cn (X.L.); 2 State Key Laboratory of Integrated Service Networks, Xidian University, Xi’an 710071, China; hanma@stu.xidian.edu.cn

**Keywords:** data center networks, cloud computing, coflow, flow scheduling, deadline

## Abstract

Many emerging Internet of Things (IoT) applications deployed on cloud platforms have strict latency requirements or deadline constraints, and thus meeting the deadlines is crucial to ensure the quality of service for users and the revenue for service providers in these delay-stringent IoT applications. Efficient flow scheduling in data center networks (DCNs) plays a major role in reducing the execution time of jobs and has garnered significant attention in recent years. However, only few studies have attempted to combine job-level flow scheduling and routing to guarantee meeting the deadlines of multi-stage jobs. In this paper, an efficient heuristic joint flow scheduling and routing (JFSR) scheme is proposed. First, targeting maximizing the number of jobs for which the deadlines have been met, we formulate the joint flow scheduling and routing optimization problem for multiple multi-stage jobs. Second, due to its mathematical intractability, this problem is decomposed into two sub-problems: inter-coflow scheduling and intra-coflow scheduling. In the first sub-problem, coflows from different jobs are scheduled according to their relative remaining times; in the second sub-problem, an iterative coflow scheduling and routing (ICSR) algorithm is designed to alternately optimize the routing path and bandwidth allocation for each scheduled coflow. Finally, simulation results demonstrate that the proposed JFSR scheme can significantly increase the number of jobs for which the deadlines have been met in DCNs.

## 1. Introduction

The last decade has witnessed explosive growth in the number of devices and data traffic from the Internet of Things (IoT). Cloud computing can provide flexible and scalable computing, storage, and networking resources in an on-demand, pay-as-you-go service model, which makes it more convenient for users to deploy and maintain various IoT applications [1]. For most IoT solutions, devices collect and send massive raw data to cloud data centers for storage, analysis, and decision making. Many emerging IoT applications have strict latency requirements or deadline constraints, such as for virtual reality, augmented reality, smart homes, smart cities, smart energies, and smart vehicles [2]. Keeping low latency and meeting deadlines is vital for both the users’ quality of service (QoS) and the service providers’ revenue in these delay-stringent IoT applications. Taking an intelligent monitoring service for example, if the response times for user requests are too long, users will resubmit or just give up their requests. If so, the abandoned overdue responses will degrade the user experience and waste the computing and communication resources. According to the statistics in [3], a 100 ms latency increase generates a 1% income loss at Amazon, while a 400 ms delay increase in search responses can reduce search volume by 0.7% for Google.

Offloading computation-intensive and delay-sensitive tasks to edge data centers is an efficient way to reduce the end-to-end communication delays in IoT applications [4,5]. However, edge computing is unable to cut down the processing time of IoT tasks in data centers. Though infrastructure providers continually updates the CPU and storage capacity of commercial servers and the link bandwidth of switches, network resources still have a performance bottleneck when handling cloud computing tasks due to the distributed nature of parallel computing and the massive communication demands among servers within data centers [6]. Popular cluster computing frameworks such as MapReduce [7] and Spark [8] have been widely applied to process the explosively growing data volume. In those typical cloud computing architectures, a cloud computing task, or a job, is usually divided into multiple stages, and each stage also includes several computing tasks that are parallel processed on different physical servers. In the cluster computing manner, the output data resulting from upper-stage computing tasks are exactly the necessary inputs for the lower-stage computing tasks, and thus a large quantity of intermediate data should be exchanged among different physical servers in a data center via data center networks (DCNs). Further, a lower-stage computing task shall not begin unless the result data from all of its upper-stage tasks have been completely delivered. It is estimated that parallel intermediate data transfers account for 33–50% of whole-job completion times [9]. Therefore, improving DCN transmission efficiency is critical for reducing the processing delays of cloud computing jobs.

Some researchers have studied deadline-aware flow scheduling in DCNs [10,11,12,13,14]. However, due to the distributed nature of cloud computing, these traditional deadline-aware scheduling schemes for individual flows are no longer applicable. After analyzing the communication patterns of typical cluster applications, Chowdhury et al. found that computing machines are grouped according to job stages or functionality, and concurrent intermediate flows between two adjacent computing stages usually have associated semantics and a collective objective [15]. This semantically related flow collection between two computing machine groups is referred to as coflow. According to the manner of cluster computing, a coflow’s completion time depends on the completion time of the last flow [16]. Thus, traditional flow-level scheduling schemes are inefficient for reducing the completion times or meeting the deadline of coflows. Further, since one cloud computing job usually contains multistage computing tasks and coflows, the dependency relationships among coflows will influence the final completion time of the job, which should be carefully considered in job scheduling. Moreover, a DCN can provide multiple routing paths between each server pair to guarantee connection reliability in typical Fat-Tree or Spine-Leaf architectures [17], and thus routing is another vital factor for reducing the job completion time for cloud data centers.

For cluster computing applications, existing deadline-aware flow scheduling schemes can be roughly classified into three types: single-stage coflow scheduling, multi-stage coflow scheduling, and joint flow scheduling and routing. In the first type, researchers usually treat different coflows as multiple independent entities and focus on meeting the deadline of each coflow separately. Chowdhury et al. modeled the inter-coflow scheduling problem as a concurrent open-shop scheduling problem and proposed heuristic algorithms to minimize the coflow completion time (CCT) or to maximize the number of deadline-guaranteed coflows [18]. They designed the Smallest Effective Bottleneck First (SEBF) algorithm and the Minimum Allocation for Desired Duration (MADD) algorithm to perform inter-coflow scheduling and intra-coflow scheduling, respectively. The SEBF algorithm calculates the predicted CCTs of all coflows and preferentially schedules the coflow with the minimum predicted CCT; the MADD algorithm determines the transmitted rates of all flows in the same coflow so that they shall have the same completion time. Chowdhury et al. also developed the coordinated coflow scheduler Varys and provided coflow APIs for cluster computing frameworks. S. Ma et al. proposed the deadline-driven coflow scheduling framework Chronos [19], which allocates bandwidth proportionally to the selected flows and reserves some residual bandwidth for unselected coflows. Therefore, Chronos is more work-conserving and starvation-free than Varys and can avoid complicated coflow admission control. S. Luo et al. realized that the deadline-missed coflow minimization problem is equivalent to the late job minimization problem in a concurrent open shop [20,21]. Thus, on the basis of concurrent open-shop scheduling solutions, they designed a decentralized, deadline-driven, coflow-aware scheduling (D${}^{2}$-CAS) system. The deadline-aware coflow scheduling schemes in [18,19,20,21] all assume that prior knowledge of coflows, such as the number, size, and endpoint of each flow, can be obtained before scheduling, but in many practical cases, detailed coflow information is not known or is incomplete [22]. Faced with this problem, researchers have proposed some online information-agnostic coflow scheduling schemes [22,23,24] in which they deduce each coflow’s remaining transmission time and divide coflows into different priority queues according to the known attributes or arrived bytes. In the second multi-stage coflow scheduling type, researchers scheduled dependent coflows from precedence-constrained job stages [25,26,27,28,29]. From the perspective of job-level performance rather than the coflow-level, meeting job deadlines is not equivalent to simply minimizing each CCT. Y. Liu et al. formulated the average job completion time (JCT) minimization problem with job deadline and coflow dependency constraints and proposed a two-level heuristic scheduling solution [25]. In their solution, the first level performs the Most Bottleneck First (MBF) algorithm to determine job orders, and the second level performs intra-job and intra-coflow scheduling while considering different coflow dependencies. W. Borjigin et al. divided dependent coflows into different stages and presented a heuristic multi-objective time-saving first (MTF) scheduling algorithm to guarantee meeting job deadlines [26]. S. Zhang formulated the multi-stage inter-coflow scheduling (MICS) problem, partitioned MICS into multiple convex single-stage inter-coflow scheduling (SICS) problems, and designed three online heuristics to balance the fairness and completion times of coflows [27]. Besides heuristic solutions, B. Tian et al. provided a deterministic approximation algorithm [28]. They formulated the multi-stage coflow scheduling problem as a weighted JCT minimization problem, relaxed it into a linear programming problem for a lower bound, and further constructed a (2M+1)-approximation scheduling algorithm, where *M* is the number of hosts. J. Wang et al. represented multi-stage jobs as directed acyclic graphs (DAGs) and proposed a genetic-algorithm-based scheduling method to reduce the time complexity while meeting job deadline demands [29].

In the third joint flow scheduling and routing type, the influence of DCN architecture is taken into consideration. The previous two scheduling types all assume that perfect traffic balancing and no over-subscription are achieved in DCNs. As a result, a DCN can be modeled as a large non-blocking switch. However, perfect traffic balancing is unrealistic, since many commercial DCN architectures adopt the equal-cost multipath (ECMP) hashing-based packet forwarding strategy [17,30]. Though some researchers have optimized flow scheduling and routing jointly to reduce the energy consumption in DCNs, they did not take the coflow communication feature into account [31]. Y. Zhao et al. were the first to jointly consider coflow scheduling and routing to optimize the average CCT, and they developed the scheduling framework RAPIER, which is compatible with commodity switches [32]. They formulated the single coflow scheduling optimization problem with routing constraints, proposed a minimum-CCT-based heuristic algorithm to determine the rate and path for each flow, and scheduled coflows with longer waiting times and less CCT as a priority. J. Jiang et al. proposed the coflow scheduler Tailor to monitor the flow bottlenecks and reroute flow to lighter load links [33]. Since RAPIER enables bandwidth preemption and Tailor enables dynamic routing, they cause frequent rerouting operations, which seems unrealistic for real-time implementation and large DCNs. Y. Li et al. designed the OneCoflow and OMCoflow algorithms to address the joint flow scheduling and routing problems for a single coflow and multiple coflows, respectively [34,35]. OneCoflow is based on convex programming and rounding and determines the routing path and bandwidth allocation for a newly arrived coflow; OMCoflow reschedules the bandwidth for each existing coflow when a new coflow arrives or when an old coflow is completed. In OneCoflow and OMCoflow, once a coflow arrives, its routing path is determined, and frequent re-routings are not allowed. Y. Chen et al. proposed the multi-hop coflow routing and scheduling (MCRS) strategy in the popular Spine-Leaf topology and allocated longer detour paths to coflows to alleviate link congestion [36,37], which is applicable to over-subscribed Spine-Leaf networks. The authors of [32,33,34,35,36,37] only focused on joint flow scheduling and routing cases at the coflow level, whereas Y. Zeng et al. were the first to study the job-level case [38]. They formulated the multi-stage job joint scheduling and routing problem as a non-linear weighted JCT minimization problem and designed a polynomial-time Multi-stage Job Scheduling (MJS) algorithm. This algorithm can achieve constant approximation ratios in various typical DCN architectures. The MJS algorithm determines the job scheduling order according to the optimal solution of the relaxed linear programming problem and schedules active jobs and coflows one by one. When a new job arrives or when a flow is completed, MJS recalculates the scheduling result until all jobs are finished. However, the assumption that a flow can be suspended in MJS is impractical and will increase the scheduling complexity.

In this paper, we aim to guarantee meeting the deadlines of as many delay-stringent IoT jobs as possible by integrating scheduling-dependent coflows and optimizing routing paths in the DCN topology. The main contributions of this paper are as follows. First, we formalize the multi-job joint scheduling and routing problem with the object of maximizing the number of jobs for which the deadlines have been met. Second, the problem is decomposed and solved by the proposed heuristic two-stage joint flow scheduling and routing (JFSR) scheme. In the first stage, the smallest relative remaining time first (SRRTF) criterion determines the scheduling order of coflows; in the second stage, the Iterative Coflow Scheduling and Routing (ICSR) algorithm calculates the rate and path allocation for each scheduled coflow. Finally, simulation results show that the proposed joint flow scheduling and routing scheme can significantly increase the number of jobs for which the deadlines have been met.

The rest of this paper is organized as follows. In Section 2, we introduce the system model, and in Section 3, we present the deadline-met job number maximization problem with coflow dependency and network constraints. The proposed two-stage JFSR scheme is introduced in Section 4. Simulation results are shown in Section 5, and Section 6 concludes the paper.

## 2. System Model

In this paper, the DCN topology is modeled as a graph G=<V,E>, where V is the node set and E is the link set. The available bandwidth of link e∈E is denoted by $B_{e}$.

The delay-stringent IoT job set to be scheduled is denoted by $J=\{J_{n},1\leq n\leq N\}$, and we assume the *n*-th job $J_{n}$ contains $N_{n}$ coflows. The *m*-th coflow of $J_{n}$ is denoted by $C_{n,m}$, and the coflow set of $J_{n}$ is denoted by $C_{n}=\left\{C_{n,m},1\leq m\leq N_{n}\right\}$. We also assume that coflow $C_{n,m}$ contains $N_{n,m}$ flows, and the flow set of $C_{n,m}$ is represented by $F_{n,m}=\left\{F_{n,m,k},1\leq k\leq N_{n,m}\right\}$. The *k*-th flow in $C_{n,m}$ is further defined as $f_{n,m,k}=<s_{n,m,k},u_{n,m,k},d_{n,m,k}>$, where $s_{n,m,k}$, $u_{n,m,k}$, and $d_{n,m,k}$ respectively represent the source node, destination node, and data volume of flow $f_{n,m,k}$. It is assumed that job information, including source nodes, destination nodes, data volumes of all coflows, and arrival times and deadlines of jobs, can be obtained once the job arrives. Note that a job’s arrival time only represents the arrival time of its first coflow, and the arrival times of subsequent coflows depend on the completion times of their upstream coflows. We assume that a coflow being transmitted cannot be preempted by other coflows, and the residual bandwidth information for each link is available to the central job scheduler whenever needed. The notations to be used are listed in Table 1.

**Figure 1:**
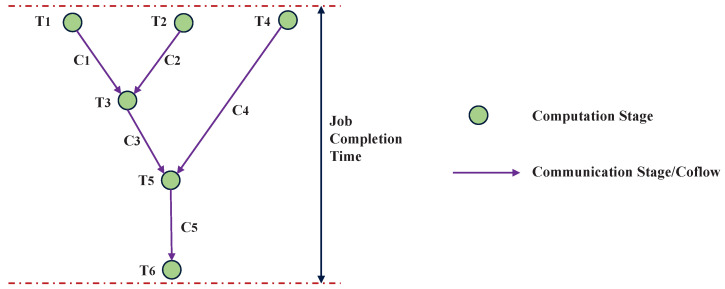
A job example represented by the DAG model; the job consists of multiple computation stages and communication stages.

In this paper we focus on the *starts*–*after* type coflow dependency, where the downstream coflow can not start before the upstream coflow ends, and a computer stage exists between them [22]. To illustrate the relationship between multi-stage jobs and coflows, a DAG-based job model from [29] is shown in Figure 1; it includes six computation stages and five communication stages. The five communication stages can be also referred to as five different coflows: denoted by $C_{1}$, $C_{2}$, $C_{3}$, $C_{4}$, and $C_{5}$, respectively. The DAG can visually show the data dependencies between adjacent coflows. For example, in Figure 1, $T_{3}$ cannot start before both $C_{1}$ and $C_{2}$ have ended, and $C_{3}$ begins after $T_{3}$ ends. The job completion time is from the beginning of $T_{1}$, $T_{2}$, and $T_{4}$ to the end of $T_{6}$. Further, the durations of all computing stages are assumed to be identical.

## 3. Deadline-Guaranteed Job Number Maximization Problem Formulation

### 3.1. Motivating Example of Joint Flow Scheduling and Routing

In this subsection, a simple motivating example is given to demonstrate how combining flow scheduling and routing can improve the JCT performance compared with isolate coflow scheduling or routing.

**Figure 2:**
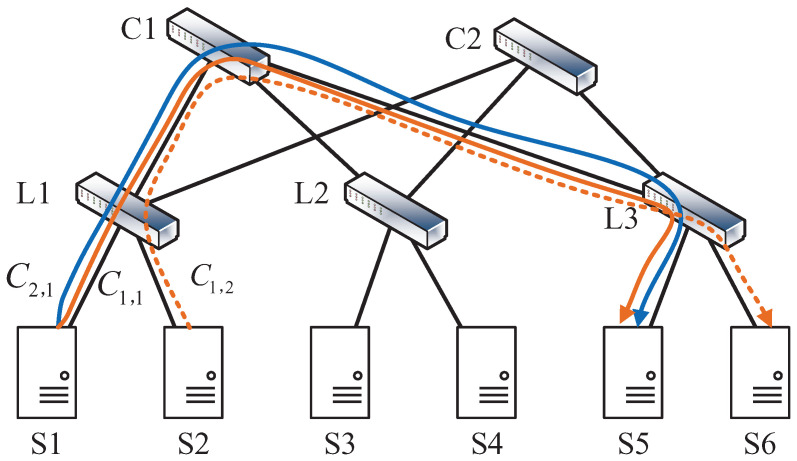
A routing case from ECMP containing coflows $C_{1,1}$, $C_{1,2}$, and $C_{2,1}$.

In the two-level Spine-Leaf DCN shown in Figure 2, two jobs are deployed. Assume job $J_{1}$ contains two coflows $C_{1,1}$ and $C_{1,2}$, and job $J_{2}$ contains coflow $C_{2,1}$. Coflows $C_{1,1}$, $C_{1,2}$, and $C_{2,1}$ are respectively represented by an orange solid line, an orange dotted line, and a blue solid line. For simplicity, we assume each coflow only contains one flow, and the sizes of $C_{1,1}$, $C_{1,2}$, and $C_{2,1}$ are, respectively, 10 MB, 50 MB, and 30 Mb. The link bandwidths are all 10 Mbps. We also assume $C_{1,1}$ and $C_{2,1}$ are sent from $S_{1}$ to $S_{5}$, and $C_{1,2}$ is sent from $S_{2}$ to $S_{6}$.

**Figure 3:**
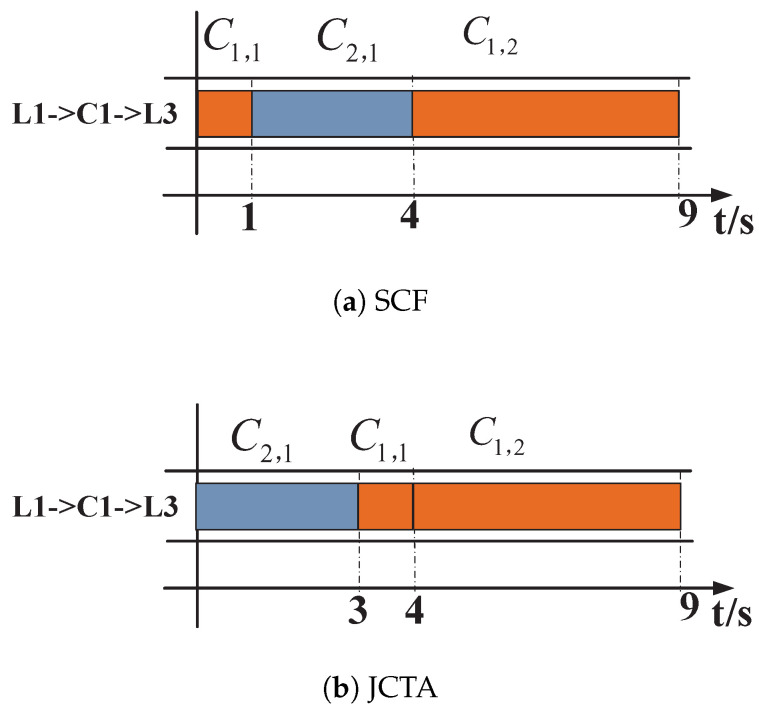
Scheduling results of SCF and JCTA strategies with the routing case above. (**a**) Completion times of $J_{1}$ and $J_{2}$ under the Smallest Coflow First (SCF) strategy. (**b**) Completion times of $J_{1}$ and $J_{2}$ under the Job Completion Time Aware (JCTA) strategy.

With a random routing policy such as equal-cost multipath (ECMP), one possible routing result is shown in Figure 2. $C_{1,1}$ and $C_{2,1}$ are transmitted via path $S_{1}-L_{1}-C_{1}-L_{3}-S_{5}$, and $C_{1,2}$ passes through $S_{2}-L_{1}-C_{1}-L_{3}-S_{6}$. In this case, the completion times of $J_{1}$ and $J_{2}$ under the Smallest Coflow First (SCF) strategy [18] and the Job Completion Time Aware (JCTA) strategy [25] are shown in Figure 3a,b, respectively. In Figure 3a, the completion times of two jobs under SCF are respectively 9 s and 4 s, while the JCTs under JCTA are respectively 9 s and 3 s in Figure 3b. Though the JCT of $J_{2}$ remains unchanged, the JCT of $J_{1}$ can be optimized by JCTA.

**Figure 4:**
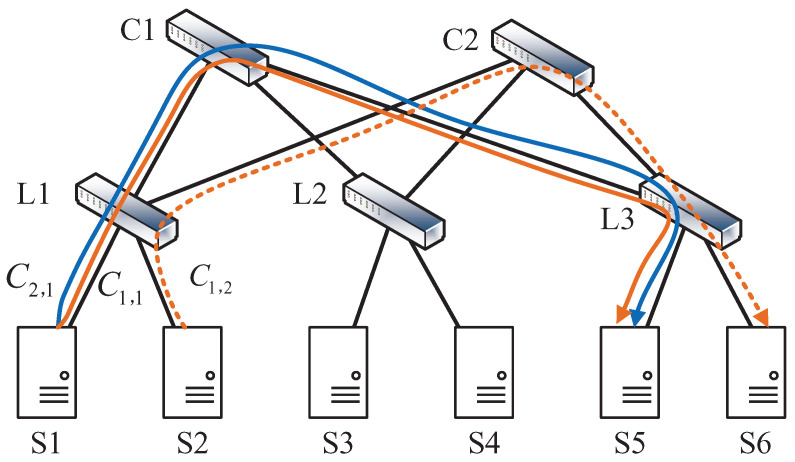
An improved routing case containing coflows $C_{1,1}$, $C_{1,2}$, and $C_{2,1}$.

**Figure 5:**
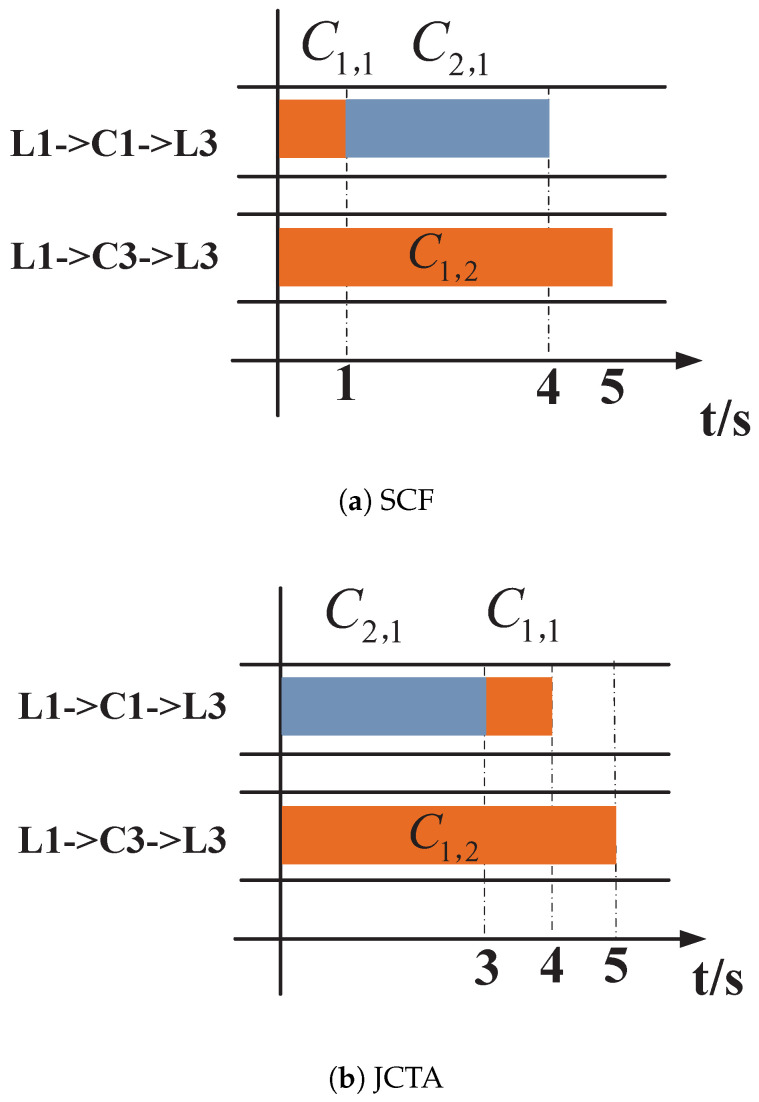
Scheduling results of SCF and JCTA strategies with the improved routing case above. (**a**) Completion times of $J_{1}$ and $J_{2}$ under the SCF strategy. (**b**) Completion times of $J_{1}$ and $J_{2}$ under the JCTA strategy.

By integrating routing and flow scheduling, an optimized routing solution is shown in Figure 4, where $C_{1,2}$ are allocated to path $S_{2}-L_{1}-C_{2}-L_{3}-S_{6}$. The corresponding scheduling results of SCF and JCTA are also shown in Figure 5a,b. In Figure 5a, the completion times of two jobs under SCF are respectively 5 s and 4 s, while the JCTs under JCTA are respectively 5 s and 3 s. Similarly, the JCT of $J_{1}$ can be reduced by JCTA.

According to the results above, the average JCT can be optimized by properly scheduling flows in the time domain after routing is determined. As shown by comparing Figure 3a,b, the SCF strategy determines the transmission sequence according to the coflow size but does not consider the coflow dependency or whether multiple coflows belong to the same job; the JCTA strategy pays more attention to the coflow dependency within the same job, which can contribute to reducing some jobs’ completion times.

Moreover, by comparing the results under the same scheduling policy but different routing paths, such as Figure 3b and Figure 5b, the JCT’s performance can be further improved by introducing the path-choosing dimension. The random routing policy may result in load unbalancing, leaving some links utilized inefficiently. As shown in Figure 2, three coflows all pass through links $L_{1}-C_{1}$ and $C_{1}-L_{3}$, making the two links the bottleneck for job processing while an idle candidate path still exists. Therefore, to guarantee meeting as many job deadlines as possible, one should properly design joint flow scheduling and routing policies rather than performing isolated optimization of flow scheduling or routing.

### 3.2. Deadline-Guaranteed Job Number Maximization Optimization Problem

We aim to maximizing the number of jobs for which the deadlines have been met, i.e.,

(1) $$\max_{}\;\sum_{n=1}^{N}y_{n}.$$

The binary variable $y_{n}$ indicates whether job $j_{n}$ can be completed before its deadline $D_{n}$, denoted by

(2) $$y_{n}=\{\begin{array}{c} 1,if\;\;T_{n}⩽r_{n}+D_{n}\\ 0,if\;\;T_{n}>r_{n}+D_{n} \end{array}.$$

Here, $r_{n}$ represents the arrival time of job $J_{n}$. The completion times of job $J_{n}$, coflow $C_{n,m}$, and flow $f_{n,m,k}$ are respectively denoted by $T_{n}$, $T_{n,m}$, and $T_{n,m,k}$. Since a job’s completion time depends on the completion time of its last coflow, and a coflow’s completion time depends on the completion time of its last flow, we have the following two completion time constraints:

(3) $$T_{n}=\max_{C_{n},m\in C_{n}}T_{n,m},\forall n$$

(4) $$T_{n,m}=\max_{f_{n,m,k}\in F_{n,m}}T_{n,m,k},\forall C_{n,m}$$

Variable $b_{n,m,k}\left(t\right)$ represents the transmission rate of flow $f_{n,m,k}$ at time *t*, and thus the traffic volume constraint for flow $f_{n,m,k}$ can be written as

(5) $$\int_{r_{n}}^{T_{n,m,k}}b_{n,m,k}\left(t\right)dt=d_{n,m,k},\forall f_{n,m,k}.$$

The *starts*–*after* dependency between the *m*-th and the $m^{\prime }$-th coflow of job $J_{n}$ is denoted by $C_{n,m^{\prime }}>C_{n,m}$, where $C_{n,m}$ begins after $C_{n,m^{\prime }}$ ends. The precedence constraint in (6) ensures that $C_{n,m}$ will not start during the transmission of $C_{n,m^{\prime }}$.

(6) $$\int_{r_{n}}^{T_{n,m^{\prime }}}b_{n,m,k}\left(t\right)dt=0,\forall C_{n,m^{\prime }}>C_{n,m},\forall f_{n,m,k}\in F_{n,m}.$$

The binary variable $x_{e}^{n,m,k}$ represents whether flow $f_{n,m,k}$ will pass through link *e*, so there are flow conservation constraints [39] as (7) and (8), where out(v) and in(v) represent the set of outgoing links from node *v* and the set of incoming links to node *v*, respectively.

(7) $$\begin{array}{c} \sum_{e\in out(v)}x_{e}^{n,m,k}-\sum_{e\in in(v)}x_{e}^{n,m,k}=0,\forall f_{n,m,k},\forall v\notin \left\{s_{n,m,k},u_{n,m,k}\right\}\; \end{array}$$

(8) $$\sum_{e\in out(s_{n,m,k})}x_{e}^{n,m,k}-\sum_{e\in in(s_{n,m,k})}x_{e}^{n,m,k}=1,\forall f_{n,m,k}$$

To guarantee that the accumulated rate of all flows allocated to link *e* will not exceed the bandwidth, we have the bandwidth constraint as

(9) $$\sum_{n}\sum_{m}\sum_{k}x_{e}^{n,m,k}b_{n,m,k}\left(t\right)⩽B_{e},\forall e,\forall t\in \left[\Gamma_{0},\Gamma_{1}\right],$$

where $\Gamma_{0}=\min_{n}r_{n}$, $\Gamma_{1}=\min_{n}\left(r_{n}+D_{n}\right)$.

Since the transmission of flow $f_{n,m,k}$ should be between the arrival time and the deadline of job $J_{n}$, we have two rate constraints as (10) and (11).

(10) $$b_{n,m,k}\left(t\right)=0,\forall f_{n,m,k},\forall t\in \left[\Gamma_{0},r_{n}\right)\cup \left[D_{n},\Gamma_{1}\right]$$

(11) $$b_{n,m,k}\left(t\right)⩾0,\forall f_{n,m,k},\forall t\in \left[r_{n},\Gamma_{1}\right)$$

Thus, the multi-job joint flow scheduling and routing problem in a DCN can be formulated as the non-linear optimization problem P in (12).

(12) $$\begin{array}{cc} P:\; & \max_{x_{e}^{n,m,k},b_{n,m,k}\left(t\right)}\;\sum_{n=1}^{N}y_{n}\\ s.t.\; & x_{e}^{n,m,k}\in \left\{0,1\right\},\forall e,\forall f_{n,m,k}\\ \; & Constraint(2)-(11) \end{array}$$

The joint scheduling and routing problem for coflows has been proved to be NP-hard [32], while P is more complicated due to the nonlinear constraints and binary variables. Therefore, we decompose P and design an alternative heuristic suboptimal solution.

## 4. Two-Stage Joint Flow Scheduling and Routing

Due to its intractability, we decompose P into two decoupled sub-problems: the inter-coflow scheduling problem and the intra-coflow scheduling problem, and we develop a two-stage JFSR scheme. In JFSR, the first stage performs inter-coflow scheduling based on the relative remaining time (RRT) criterion and determines the target coflow to be further processed in the second stage. The second stage aims to figure out whether the target coflow can be scheduled immediately by arranging the routing path and transmission rate for each flow in the target coflow with the proposed Iterative Coflow Scheduling and Routing (ICSR) algorithm. For simplicity, we made three assumptions. First, once a coflow has been scheduled, the routing paths and transmission sequences of its flows cannot be preempted by any other coflow that is scheduled later. Second, the bandwidth allocated to each flow remains constant during its transmission. Third, each flow is unsplittable, i.e., it is not allowed to be divided into several segments and transmitted via different paths.

### 4.1. The Smallest Relative Remaining Time First Criterion

In the first stage of JFSR, the key is to determine the target coflow to be scheduled at time $t_{0}$. In summary, there are three times when coflows will be checked for possible scheduling. The first when a new job arrives, and thus the system immediately investigates whether its first-stage coflows do not have any *starts*–*after*-type dependencies from upstream coflows and can thus be scheduled via the following ICSR algorithm in the second stage. If granted, a first-stage coflow from a newly arrived job can be transmitted at once; if denied due to limited bandwidth, the first-stage coflow will be put on the waiting list to wait for the next scheduling chance.

The second kind of schedule timing is when a coflow from a previously arrived job is ready, i.e., when all of its upstream computation stages are finished. Similarly, the system checks whether this coflow can begin its transmission or should go on the waiting list.

The third kind of schedule timing is when a coflow from a previously arrived job is finished and some bandwidth resource is released. In this case, the system investigates whether any queuing coflow on the waiting list can have a chance. In this case, the scheduling priority of a queuing coflow is determined by the SRRTF criterion. If coflow $C_{n,m}$ is on the waiting list, job $J_{n}$ has $G_{n}$ unscheduled coflow stages at time $t_{0}$, and coflow $C_{n,m}$ belongs to the *g*-th stage, then the RRT of coflow $C_{n,m}$ at time $t_{0}$ is defined as $R_{n,m}=g\left(r_{n}+D_{n}-t_{0}\right)/G_{n}$. For consistency, the RRT values of ready-to-go coflows from the first two kinds of timings are all set as zero. As a result, in all the three kinds of timings, ready-to-go coflows and queuing coflows all update their RRT values, and the coflow with the minimum RRT is chosen as the most urgent one and is handled by the second stage.

### 4.2. The Iterative Coflow Scheduling and Routing Algorithm

In the second stage, we focus on joint scheduling and routing for the single coflow chosen from the first stage. If coflow $C_{n,m}$ is chosen, problem P is simplified into P1, which aims to minimize the CCT of $C_{n,m}$. In P1, (14)–(17) respectively represent the binary variable constraint, rate variable constraint, traffic volume constraint, and bandwidth constraint, while (18) and (19) denote the flow conservation constraints.

(13) $$\begin{array}{cc} P1:\; & \min_{b_{n,m,k},x_{e}^{n,m,k}}\;T_{n,m} \end{array}$$

(14) $$\begin{array}{cc} s.t.\; & x_{e}^{n,m,k}\in \left\{0,1\right\},\forall e,\forall f_{n,m,k}\in F_{n,m} \end{array}$$

(15) $$\begin{array}{cc} \; & b_{n,m,k}⩾0,\forall f_{n,m,k}\in F_{n,m} \end{array}$$

(16) $$\begin{array}{cc} \; & T_{n,m}⩾\frac{d_{n,m,k}}{b_{n,m,k}},\;\;\;\;\;\;\;\;\forall f_{n,m,k}\in F_{n,m} \end{array}$$

(17) $$\begin{array}{cc} \; & \sum_{f_{n,m,k}\in F_{n,m}}x_{e}^{n,m,k}b_{n,m,k}⩽B_{e},\forall e \end{array}$$

(18) $$\begin{array}{cc} \; & \sum_{e\in out(v)}x_{e}^{n,m,k}=\sum_{e\in in(v)}x_{e}^{n,m,k},\forall f_{n,m,k}\in F_{n,m},\forall v\notin \left\{s_{n,m,k},u_{n,m,k}\right\} \end{array}$$

(19) $$\begin{array}{cc} \; & \sum_{e\in out(s_{n,m,k})}x_{e}^{n,m,k}-\sum_{e\in in(s_{n,m,k})}x_{e}^{n,m,k}=1,\forall f_{n,m,k}\in F_{n,m} \end{array}$$

In fact, P1 is an integer multi-commodity flow problem, which has been proved to be NP-hard [32]. Therefore, based on the alternating optimization principle, an Iterative Coflow Scheduling and Routing (ICSR) algorithm is proposed to alternately update the bandwidth and path allocation for the coflow being scheduled. The basic idea of ICSR is to fix one of the two kinds of variables—the bandwidth allocation variable $b_{n,m,k}$ or the path allocation variable $x_{e}^{n,m,k}$—in turns and solve for the other one.

At first, the flow rate $b_{n,m,k}$ is initialized to obtain the candidate paths. For flow $f_{n,m,k}$, its initial rate is set as $b_{n,m,k}\left(0\right)=d_{n,m,k}/\left(r_{n}+D_{n}-t_{0}\right)$. By treating the bandwidth allocation variable $b_{n,m,k}$ as constant and relaxing the binary constraint of the path allocation $x_{e}^{n,m,k}$, P1 is simplified into the feasibility problem P2. P2 is a linear programming (LP) problem and can be efficiently solved. Though the object of P2 is a constant, a feasible solution of P2 is a group of routing paths for which the available bandwidths can satisfy the bandwidth constraints. When the available bandwidth is limited, it is possible that the value of $x_{e}^{n,m,k}$ obtained from P2 is a fraction. In this case, flow $f_{n,m,k}$ can be split into multiple sub-flows, which are transmitted through different paths. Since each flow is unsplittable in our fundamental assumption, at the end of the ICSR algorithm, the values of $x_{e}^{n,m,k}$ should be recovered to be binary.

During the first iteration loop, it is possible that P2 has no feasible solutions. In this case, there does not exist a candidate group of routing paths with adequate available bandwidth to accommodate coflow $C_{n,m}$. Thus, the ICSR algorithm ends and coflow $C_{n,m}$ is put on the waiting list. When a previously scheduled coflow is finished and the bandwidth is released, all coflows on the waiting list update their RRT values and wait to be processed by the two stages of JFSR again.

(20) $$\begin{array}{cc} P2:\; & \min_{x_{e}^{n,m,k}}\;0\\ s.t.\; & 0⩽x_{e}^{n,m,k}⩽1,\forall e,\forall f_{n,m,k}\in F_{n,m}\\ \; & Constraint(17)–(19) \end{array}$$

With the feasible solutions of $x_{e}^{n,m,k}$ obtained from P2, we calculate the transmission rate $b_{n,m,k}$. By treating $x_{e}^{n,m,k}$ as constant and introducing the auxiliary variable $a=1/T_{n,m}$, P1 is simplified and reformulated into the LP problem P3. The resulting values of $b_{n,m,k}$ will be the input of P2 in the next iteration.

(21) $$\begin{array}{cc} P3:\; & \max_{b_{n,m,k}}\;a\\ s.t.\; & b_{n,m,k}⩾0,\forall f_{n,m,k}\in F_{n,m}\\ \; & b_{n,m,k}⩽d_{n,m,k}a,\forall f_{n,m,k}\in F_{n,m}\\ \; & \sum_{f_{n,m,k}\in F_{n,m}}x_{e}^{n,m,k}b_{n,m,k}⩽B_{e},\forall e \end{array}$$

The pseudocode of the ICSR algorithm is given in Algorithm 1. At first, the path allocation variable $x_{e}^{n,m,k}$ and the rate allocation variable $b_{n,m,k}$ are alternately optimized according to P2 and P3, respectively. After enough iterations, the resulting CCT may be satisfying, but there is a high probability that the values of $x_{e}^{n,m,k}$ are not binary. To ensure $x_{e}^{n,m,k}$ is binary, which guarantees each flow only goes through one path, for flow $f_{n,m,k}$ we check the value of $w_{n,m,k}^{i}=\min_{e\in P_{n,m,k}^{i}}x_{e}^{n,m,k}$ among all candidate paths, where $P_{n,m,k}^{i}$ represents the *i*-th candidate path of $f_{n,m,k}$. The candidate path with the maximum value of $w_{n,m,k}^{i}$ is chosen as the final transmission path, denoted by $P_{n,m,k}^{*}$. Thereafter, the final transmission rate of flow $f_{n,m,k}$, denoted by $b_{n,m,k}^{*}$, and the corresponding CCT of $C_{n,m}$, denoted by $T_{n,m}^{*}$, are determined by solving P3 again with the recovered binary $x_{e}^{n,m,k}$. Thus, the joint scheduling for the finishing and routing of coflow $C_{n,m}$ is finished. As time goes by, the system continues to repeat the two steps of the JSFR scheme.
**Algorithm 1** Iterative Coflow Scheduling and Routing Algorithm**Require:** The prior information of coflow $C_{n,m}$.**Ensure:** The CCT, path, and rate allocation of $C_{n,m}$.1:Initialization: Set iteration number n=0, $b_{n,m,k}\left(0\right)=d_{n},m,k/\left(r_{n}+D_{n}-t_{0}\right)$ for all flows in $C_{n,m}$.2:**if** P2 is infeasible **then**3:      Put $C_{n,m}$ on the waiting list.4:**else**5:      **repeat**6:         1) Update $x_{e}^{n,m,k}\left(n+1\right)$ for all flows in $C_{n,m}$ and all links by solving P2 with known $b_{n,m,k}\left(n\right)$.7:       2) Update $b_{n,m,k}\left(n+1\right)$ for all flows in $C_{n,m}$ by solving P3 with known $x_{e}^{n,m,k}\left(n+1\right)$, and n←n+1.8:      **until** the predetermined iteration number.9:      For flow $f_{n,m,k}$, choose the final path $P_{n,m,k}^{*}=\arg\max\min_{e\in P_{n,m,k}^{i}}x_{e}^{n,m,k}$, and set $x_{e}^{n,m,k}=1,\forall e\in P_{n,m,k}^{*}$, and $x_{e}^{n,m,k}=0,\forall e\notin P_{n,m,k}^{*}$.10:  Calculate the final rate $b_{n,m,k}^{*}$ and CCT $T_{n,m}^{*}$ by solving P3 with the final $x_{e}^{n,m,k}$.11:**end if**

## 5. Simulation Results

In this section, the validity of the proposed JFSR scheme is confirmed by Monte Carlo simulation. The Fat-Tree topology with k=4 is adopted as the DCN topology [40], and we utilize Pulp as the LP problem solver [41]. Job arrivals follow the Poisson process with an average arrival interval λ. The longest acceptable job duration, the number of coflows in each job, and the number of flows in each coflow all obey uniform distribution. The data size of flow $f_{n,m,k}$ follows a Gaussian distribution as $d_{n,m,k}∼N\left(100,30\right)$ Mb. The link bandwidth $B_{e}$ is set as 10 Gbps.

The performance of three scheduling schemes were simulated and compared, i.e., the Baseline scheme, the Scheduling-Only scheme, and the proposed JFSR scheme. The Baseline scheme adopted the ECMP routing strategy, shared the bandwidth fairly, and scheduled coflows based on the SCF policy; the Scheduling-Only scheme also adopted ECMP and fair bandwidth sharing but scheduled coflows according the SRRTF criterion.

**Figure 6:**
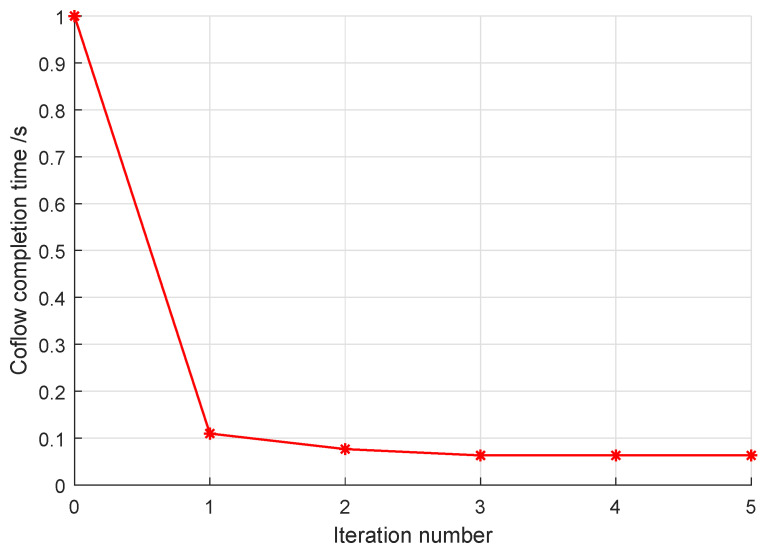
Convergence performance of ICSR for a single coflow instance.

At first, we examine the convergence property of ICSR. The iterative CCT values in one coflow realization are shown in Figure 6. There are 130 flows in this coflow, and its RRT is set as 1 s. As shown in Figure 6, the CCT gradually approaches the minimal value. It should be noticed that this smooth convergence is only guaranteed in the iteration steps of ICSR. After its last iteration step, the ICSR algorithm recovers the binary variable $x_{e}^{n,m,k}$, which may not be optimal for the relaxed version of P1. However, the low-complexity iterative searching of ICSR allows for the performance of more iterative steps and may capture a satisfactory solution within the limited decision time.

**Figure 7:**
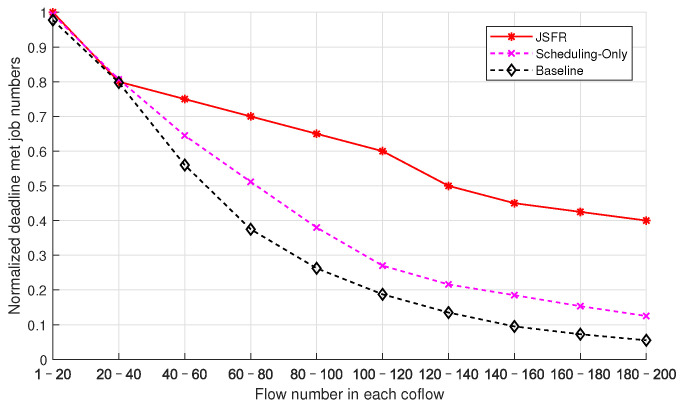
The average normalized numbers of jobs for which the deadlines have been met with the increase in the flow number in each coflow under different scheduling policies.

The performance metric used to measure the deadline-guarantee capability of scheduling schemes in the simulation is the average normalized number of jobs for which the deadlines have been met, which is defined as the mean of the ratios of the number of jobs for which the deadlines have been met to the total number of all jobs in different snapshots with the same simulation parameters. The average normalized numbers of jobs for which the deadlines have been met with different flow numbers in each coflow from three scheduling schemes are compared in Figure 7. The average job arrival interval is λ = 3s, the longest acceptable job duration $D_{n}$ is uniformly distributed in the interval [10s,50s], and the coflow number of each job is subject to a discrete uniform distribution in the interval [10,20]. The flow number in each coflow is also subject to a discrete uniform distribution, and the end points of the distribution interval increase with a step size of 20 until [180,200] in different simulation cases. As shown in Figure 7, when the flow number in each coflow is below 20, the average normalized numbers of jobs for which the deadlines have been met from the three scheduling schemes are close to one. In this case, the flow load is relatively light and almost all jobs’ deadlines can be met. As the flow number in each coflow gradually increases, the proposed JFSR scheme achieves significantly better performance than the other two scheduling schemes, while the Scheduling-Only scheme also outperforms the Baseline scheme. Therefore, integrating optimizing the routing path into coflow scheduling can efficiently guarantee the deadline-met performance for multi-stage time-sensitive jobs. The Baseline and Scheduling-Only schemes both adopt ECMP as the random routing strategy and use fair bandwidth sharing, and the hash-table-based path selection may arrange for too many flows to share the bandwidth of the same bottlenecked link. As a result, the limited bandwidth allocated to every flow passing through the bottlenecked link will remarkably increase the completion times of these unlucky flows, which will further raise the completion times of the coflows as well the jobs that contain these delayed flows. As the flow number in each coflow increases, the effect becomes more and more pronounced. At the same time, the proposed JFSR scheme can optimize the routing paths and the bandwidth allocations for the flows of each coflow via the designed ICSR algorithm. Thus, the completion time of each coflow can be reduced, which will further contribute to reducing the JCT. Therefore, the increase in each coflow’s flow number has a smaller impact on the curve of JFSR in Figure 7. Further, the performance difference between the Baseline and Scheduling-Only schemes can be attributed to their inter-coflow scheduling policies. The Baseline scheme determines the scheduling priorities of coflows according to their data volumes, i.e., via the SCF policy, but does not involve the deadline information. The Scheduling-Only scheme considers prior deadline information of jobs as well as the RRT of coflows and thus can guarantee more jobs’ deadlines are met than the Baseline scheme.

**Figure 8:**
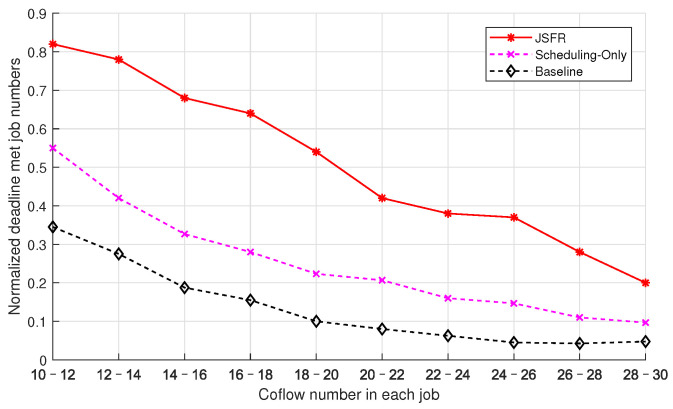
The average normalized numbers of jobs for which the deadlines have been met with the increase in the coflow number in each job under different scheduling policies.

The influence of the coflow number in each job on the average normalized number of jobs for which the deadlines have been met from three scheduling schemes is illustrated in Figure 8. The settings of λ and $D_{n}$ are identical as those in Figure 7, and the flow number in each coflow is subject to a discrete uniform distribution on the interval [80,100]. The coflow number in each job is also subject to a discrete uniform distribution, and the end points of the distribution interval increase from [10,12] to [28,30] in different simulation cases. As the coflow number in each job increases, the average normalized numbers of punctual jobs from the three scheduling schemes all gradually decrease in Figure 8. Similar to Figure 7, the proposed JFSR scheme can still guarantee meeting many more job deadlines than the other two scheduling schemes, and the Scheduling-Only scheme is also superior to the Baseline scheme in Figure 8. With the increase in the coflow number in each job, heavier flow loads are deployed into the network, and the *starts*–*after*-type coflow dependency relationships becomes more and more complicated. The JFSR scheme can both optimize the completion of each coflow via the ICSR algorithm and arrange for a more appropriate dispatching sequence for coflows via the SRRTF criterion. These two factors ensure the JFSR scheme can maintain better performance at guaranteeing meeting job deadlines than the other two scheduling schemes under different flow loads.

## 6. Conclusions

In this paper we focused on how to guarantee meeting the deadlines of time-sensitive IoT jobs by jointly considering flow scheduling and routing in DCNs. First, the multi-job joint flow scheduling and routing problem was formulated as a non-linear optimization problem with the object of maximizing the number of jobs for which the deadlines have been met. Then, we decomposed the problem into two inter-coflow scheduling and intra-coflow scheduling sub-problems to reduce the complexity of solving the problem. In the inter-coflow scheduling subproblem, coflows are ordered by their relative remaining times; in the intra-coflow scheduling subproblem, an iterative coflow scheduling and routing (ICSR) algorithm was designed to determine the transmission rates and routing paths for the scheduled coflow. Simulation results verified the proposed two-stage joint flow scheduling and routing can efficiently improve the number of jobs for which the deadlines have been met in DCNs. However, the proposed JFSR scheme is based on some ideal assumptions, and these assumptions are less practical in real data center scenarios. For example, we assume that complete job information, including the source nodes, destination nodes, and data volumes of all coflows in each job, can be obtained once the job arrives, and the residual bandwidth information for each link is available whenever needed. In fact, in practical scenarios, the detailed coflow information is not known or is incomplete, and the link’s residual bandwidth information is usually outdated. Even so, we think the basic principle of the proposed JFSR scheme is still applicable for designing online information-agnostic coflow scheduling schemes and may be helpful for researchers or engineers in this field. For example, incomplete coflow information can be replaced by statistical information derived from massive historical records of the same kind of cluster computing jobs, and link residual bandwidth may be obtained by advanced forecasting algorithms. In the future, these realistic constraints should be considered for designing a more practical flow scheduling scheme for cloud data centers, which is attractive for researchers or engineers developing software programs to configure and manage physical or virtual network resources for cloud data center infrastructure providers or service providers.

## Figures and Tables

**Table 1:** Notations of variable and constants.

Symbol	Definition
V	Node set
E	Link set
$B_{e}$	Bandwidth of link *e*
J	Job set
*N*	Number of jobs
$C_{n}$	Coflow set of job $J_{n}$
$N_{n}$	Number of coflows in job $J_{n}$
$F_{n,m}$	Flow set of coflow $C_{n,m}$
$N_{n,m}$	Number of flows in coflow $C_{n,m}$
$s_{n,m,k}$	Source node of flow $f_{n,m,k}$
$u_{n,m,k}$	Destination node of flow $f_{n,m,k}$
$d_{n,m,k}$	Data volume of flow $f_{n,m,k}$
$r_{n}$	Arrival time of job $J_{n}$
$D_{n}$	Acceptable longest duration of job $J_{n}$
$T_{n}$	Completion time of job $J_{n}$
$T_{n,m}$	Completion time of coflow $C_{n,m}$
$T_{n,m,k}$	Completion time of flow $f_{n,m,k}$
$b_{n,m,k}\left(t\right)$	bandwidth of flow $f_{n,m,k}$ at time *t*
$G_{n}$	Number of remaining coflow stages of job $J_{n}$
$R_{n,m}$	Relative remaining time of coflow $C_{n,m}$
$P_{n,m,k}^{i}$	*i*-th candidate path of $f_{n,m,k}$

## Data Availability

Data are contained within the article.
